# Development of a New Positron Emission Tomography Tracer for Targeting Tumor Angiogenesis: Synthesis, Small Animal Imaging, and Radiation Dosimetry

**DOI:** 10.3390/molecules18055594

**Published:** 2013-05-15

**Authors:** Cam Patterson, C. Brandon Frederick, Hong Yuan, Laura A. Dyer, Pamela Lockyer, David S. Lalush, Anka N. Veleva

**Affiliations:** 1 McAllister Heart Institute, University of North Carolina, Chapel Hill, NC 27599, USA; E-Mails: cam_patterson@med.unc.edu (C.P.); ldyer1@email.unc.edu (L.A.D.); pamela_lockyer@med.unc.edu (P.L.); 2 Department of Medicine, University of North Carolina, Chapel Hill, NC 27599, USA; 3 Department of Cell and Developmental Biology, University of North Carolina, Chapel Hill, NC 27599, USA; 4 Department of Pharmacology, University of North Carolina, Chapel Hill, NC 27599, USA; 5 Department of Biomedical Engineering, North Carolina State University, Raleigh, NC 27695, USA; E-Mails: cbfreder@email.unc.edu (C.B.F); dslalush@ncsu.edu (D.S.L.); 6 Biomedical Research Imaging Research Center, University of North Carolina at Chapel Hill, NC 27599, USA; E-Mail: hong_yuan@med.unc.edu

**Keywords:** development of new ^64^Cu radiolabeled peptide, radiopharmaceuticals, targeted radioconjugates, positron emission tomography (PET) imaging, tumor angiogenesis, tumor aggressiveness, responses to therapy

## Abstract

Angiogenesis plays a key role in cancer progression and correlates with disease aggressiveness and poor clinical outcomes. Affinity ligands discovered by screening phage display random peptide libraries can be engineered to molecularly target tumor blood vessels for noninvasive imaging and early detection of tumor aggressiveness. In this study, we tested the ability of a phage-display-selected peptide sequence recognizing specifically bone marrow- derived pro-angiogenic tumor-homing cells, the QFP-peptide, radiolabeled with ^64^Cu radioisotope to selectively image tumor vasculature *in vivo* by positron emission tomography (PET). To prepare the targeted PET tracer we modified QFP-phage with the DOTA chelator and radiolabeled the purified QFP-phage-DOTA intermediate with ^64^Cu to obtain QFP-targeted radioconjugate with high radiopharmaceutical yield and specific activity. We evaluated the new PET tracer *in vivo* in a subcutaneous (s.c.) Lewis lung carcinoma (LLC) mouse model and conducted tissue distribution, small animal PET/CT imaging study, autoradiography, histology, fluorescence imaging, and dosimetry assessments. The results from this study show that, in the context of the s.c. LLC immunocompetent mouse model, the QFP-tracer can target tumor blood vessels selectively. However, further optimization of the biodistribution and dosimetry profile of the tracer is necessary to ensure efficient radiopharmaceutical applications enabled by the biological specificity of the QFP-peptide.

## 1. Introduction

Angiogenesis is a hallmark of cancer and is generally associated with aggressive cancer growth and poor patient prognosis. Tumors form new blood vessels either from pre-existing mature ones or *de novo* by recruiting circulating endothelial and hematopoietic precursor cells [1]. Stromal cells of bone marrow origin have been identified in the vasculature of several pre-clinical models [2,3,4]. As shown in a bone marrow transplant model with Id1+/-Id3-/- tumor-resistant mutant mice, transplanted bone marrow endothelial and hematopoietic precursor cells are fully capable of supporting tumor growth [5]. In humans, bone marrow-derived endothelial cells have been detected in patients with multiple myeloma [6], primary breast cancer [7], non-small cell lung cancer [8], and malignant gliomas [9]. According to a report by the Voest group [10] increased levels of immature precursors in the peripheral blood of patients with breast, colon, prostate, head and neck, renal and ovarian cancer correlate with aggressive disease. Furthermore, circulating progenitor cells of bone marrow origin have been exploited as a potential biomarker to guide the applications of antiangiogenic therapy in cancer patients [11]. Together, these studies suggest that circulating tumor-homing cells participate in tumor angiogenesis and represent a valuable target for development of diagnostic and therapeutic agents with improved tumor selectivity.

Screening of phage display random peptide libraries has emerged as a powerful approach for the discovery of new peptide ligands that can bind with high affinity and specificity to a variety of targets including angiogenic blood vessels [12,13,14,15]. When radiolabeled, such phage-display-derived peptides hold promise as molecularly targeted imaging agents for noninvasive tumor characterization and early assessment of responses to therapy. Although antibodies and proteins have been considered as targeted imaging agents, these high molecular weight biologicals demonstrate poor accumulation and penetration in solid tumors, slow *in vivo* clearance, and sensitivity to labeling conditions. To overcome these limitations, development efforts have focused on smaller moieties such as peptides. In addition to good transport properties, and the ability to both penetrate rapidly into solid tumors and recognize hidden or uncommon epitopes, compared to antibodies and antibody fragments, peptides have low toxicity and immunogenicity, and can be synthesized in large quantities at low cost. In parallel to screening peptide libraries, combinatorial strategies have been devised for selection of engineered affinity protein scaffolds. A protein scaffold utilizes a protein framework that can carry altered amino acid residues to impart new binding specificity [16]. The most thoroughly investigated and successful protein scaffolds include: affibody molecules [17], adnectins [18], anticalins [19], peptide aptamers [20], avimers [21], and darpins [22]. Because of their inherent target specificity peptide ligands and engineered affinity proteins are considered highly promising for *in vivo* diagnostics as imaging agents. A radiolabeled molecular agent can specifically target disease-associated marker and this interaction can be visualized *in vivo* noninvasively by gamma- or positron emission tomography (PET).

Molecular PET is a highly sensitive imaging modality that relies on the delivery to a tissue or organ of interest of a targeted probe containing a positron-emitting radionuclide. Several radioisotopes, including radiometals have been developed for use in PET imaging in conjunction with biological targeting vectors [23,24]. Copper radioisotope, ^64^Cu, has the advantage of emitting very low energy positrons, with an average energy of 0.28 MeV and maximum β+ energy of 0.655 MeV. Because the maximum β+ energy of ^64^Cu is almost identical to the energy of the clinically validated PET nuclide, ^18^F, the resulting images are of very good quality. In addition to molecular imaging, ^64^Cu has promising applications in targeted radiotherapy as well [25,26]. Moreover, ^64^Cu can be produced via a variety of reaction pathways with either a nuclear reactor or a cyclotron. The relatively long ^64^Cu half-life of 12.7 h (as opposed to ^18^F half-life of 1.6 h) allows the ^64^Cu isotope to be distributed from a central production station, thus making it available to radio-pharmaceutical scientists and clinicians who can work with it without having in-house access to a cyclotron. 

New and innovative diagnostic imaging approaches have been developed during the past decade utilizing ^64^Cu-metallated peptides. In contrast to the most commonly used PET tracer, ^18^F-fluorodeoxyglucose (FGD), ^64^Cu-labeled peptides are more specific than FDG in oncology studies providing for noninvasive *in vivo* visualization and characterization of various receptor-expressing tumor tissues. Pioneering efforts of ^64^Cu radiochemistry and radiopharmaceutical development have been based upon bombesin (BBN) to noninvasively characterize expression of gastrin releasing peptide (GRP) receptors [27]. More recently the Smith group at the University of Missouri [28] designed and characterized new ^64^Cu-labeled bombesin targeting vector for molecular imaging studies in GRP-expressing tumor models and demonstrated high *in vivo* affinity and selectivity in prostate PC-3 tumor-bearing mouse model. Intensive work has been undertaken on the development of ^64^Cu-radiolabeled peptides for imaging integrin expression [29]. Most molecular imaging studies of integrins exploit the minimal binding amino acid sequence, Arg-Gly-Asp (RGD), of several extracellular matrix proteins, e.g., vitronectin, laminin, and fibronectin, as the lead structure to impart specificity to integrin receptors. Various design approaches to optimize the lead RGD vector for improved *in vivo* pharmacokinetics and tumor accumulation and retention have been implemented in a variety of tumor bearing mouse models. Dumont *et al*. [30] designed a ^64^Cu-RGD conjugate and characterized it as an attractive alternative to ^18^F-labeled compounds because ^64^Cu-labeled RGD tracer allowed for delayed imaging with improved tumor-to-background ratios. Another strategy to target cellular integrins is the utilization of the knottin peptide. Nielsen *et al*. [31] developed ^64^Cu-DOTA-knottin tracer and conducted head-to-head comparison with FDG in a transgenic mouse model of lung cancer. Compared to FDG, ^64^Cu-DOTA-knottin 2.5F tracer produced statistically higher tumor-to-background ratio which resulted in high-contrast, high-quality micro PET images. 

Numerous applications of ^64^Cu radionuclide have been reported for PET radioimmunoimaging and radioimmunotherapy. Antibodies that are clinically relevant, e.g., Abergin and Cetuximab, have been labeled with **^64^**Cu for PET imaging [32,33,34]. ^64^Cu-DOTA cetuximab was evaluated in mice bearing epidermal growth factor receptor (EGFR) -positive and EGFR-negative tumors for quantitative PET imaging [34]. Investigators found that **^64^**Cu-DOTA-cetuximab exhibited notable uptake in EGFR-expressing tumors and low accumulation in EGFR-negative tumors. On a clinical level the **^64^**Cu-TETA-1A3 radioimmunoconjugate was used to evaluate 36 patients with suspected primary or advanced colorectal cancer [35]. All patients had computed tomography (CT) and PET scans at 4 h p.i. and 36 h p.i. The study detected 11 new tumor sites that were previously not detected by CT or molecular resonance (MR) imaging demonstrating that PET radioimmunoimaging with **^64^**Cu may have important applications in clinical oncologic imaging especially for detection of smaller lesions that are undetected by CT or MRI. Although radioimmunoimiganing with **^64^**Cu has showed satisfactory results it is considered that **^64^**Cu seems to be more suitable candidate for imaging in conjunction with engineered targeting vectors such as affinity protein scaffolds and short peptide ligands [34].

**Figure 1:**
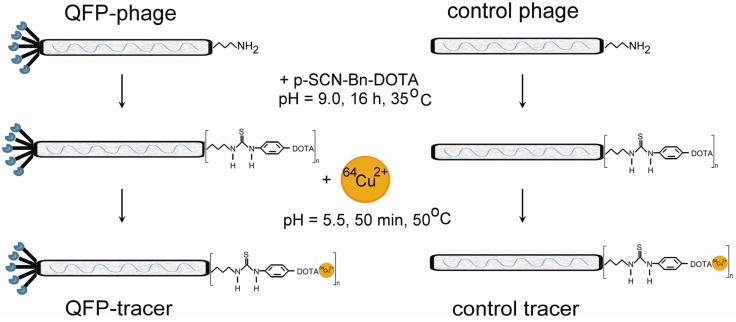
Flow chart outlining the synthetic route and the reaction conditions for preparing the QFP-targeted and the control tracers. First, the M13 phage scaffold was modified with the macrocyclic chelator DOTA by coupling the amino groups exposed on the phage surface with the bifunctional p-SCN-Bn-DOTA derivative. In a second step, the QFP-phage-DOTA or the control-phage-DOTA constructs were radiolabeled with the ^64^Cu radioisotope.

The purpose of the present study is to prepare peptide-based PET tracer radiolabeled with ^64^Cu, and to characterize its biodistribution and imaging properties *in vivo*. By screening a phage display dodecapeptide library *in vivo*, we were first to discover a novel amino acid sequence, the QFP-peptide (QFPPKLTNNSML), that specifically binds with high affinity to bone marrow-derived circulating tumor-homing cells [36] and hence the motivation to pursue further characterization. Herein, we will evaluate the ability of the QFP-peptide labeled with a positron emitter, to selectively image tumor vasculature *in vivo* by positron emission tomography (PET). We utilize the phage particle as a scaffold on which multiple copies of the QFP-peptide are displayed (see Figure 1). The phage coat proteins allow for facile orthogonal radiolabeling without interference with the binding properties of the QFP-peptide. To prepare the targeted PET tracer, QFP-phage was modified with the chelator DOTA. QFP-phage-DOTA construct was purified and radiolabeled with ^64^Cu to yield a targeted radioconjugate with high radiochemical efficiency and specific activity. *In vivo* evaluation of the targeted tracer was performed in a subcutaneously implanted Lewis lung carcinoma (LLC) model in immunocompetent C57Bl6 mice and included tissue distribution, small animal PET/CT imaging studies, autoradiography, histology, fluorescence microscopy, and dosimetry assessments. Control experiments were run in parallel with a nontargeted radiotracer and the results were compared.

## 2. Results and Discussion

### 2.1. Synthesis and Characterization of ^64^Cu-Radiolabeled Tracers

Because phage have their own *in vivo* biodistribution patterns, QFP-peptide displayed on the surface of a phage particle should be validated against a control phage construct that displays no peptide, *i.e.*, the wild type phage analog. For this reason, we synthesized the targeted QFP-phage-DOTA-^64^Cu tracer and the nontargeted, wild type -phage-DOTA-^64^Cu control and compared their properties *in vivo*.

In Figure 1, the synthetic route and the reaction conditions for preparing the targeted and the control tracers are presented. The amino groups on the phage scaffold coat protein pVIII reacted with the thioisocyanate group of the bifunctional DOTA ligand to yield a thiourea bond. The conjugation reaction was carried in carbonate-bicarbonate buffer (pH 9.0) at 35 °C overnight. Under these reaction conditions, approximately five DOTA ligands were attached per phage particle [36]. Unbound DOTA was separated by ultracentrifugation. 

For radiolabeling, the QFP-phage-DOTA or control-phage-DOTA were re-suspended in 0.1 M sodium acetate buffer (pH 5.5). ^64^CuCl_2_ solution was added, and the reactants were heated at 50 °C for 50 min. Unbound ^64^Cu was quenched with EDTA and separated from the radiolabeled tracer by ultracentrifugation. The radiochemical yield was greater than 98% and the specific activity ranged for both tracers from 20 to 50 Ci/mmol (740 to 1850 GBq/mmol) based on calculations per phage particle.

An alternative to the DOTA-Bn-SCN bifunctional chelator used in this study is the DOTA-NHS-ester derivative. Although both functional groups allow for straightforward and efficient labeling procedures there are differences in the ability of the two bifunctional chelators to form stable complexes with ^64^Cu. Compared with the only three carboxylate groups of the DOTA-NHS-ester available for complexation, the presence of four carboxylate arms and an additional benzyl functionality attached to the carbon backbone of the DOTA-Bn-SCN will produce more stable ^64^Cu-DOTA complex. Radiometal-chelator complex stability *in vivo* is of critical importance for clinical success of a metal-based tracer and should be further investigated in more detail.

### 2.2. *In Vivo* Biodistribution Studies

**Figure 2:**
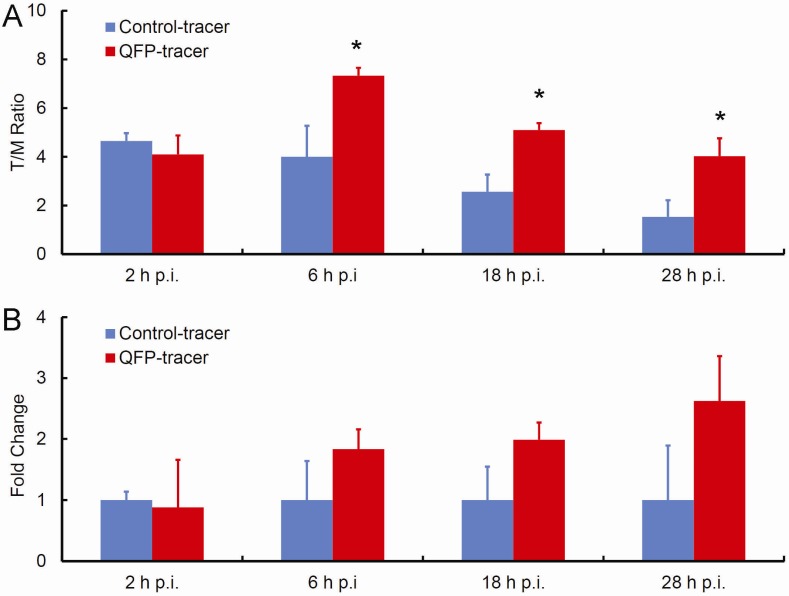
Ability of the QFP-tracer to specifically accumulate in tumor tissue based on direct tumor sampling. (**A**) Tumor-to-muscle (T/M) uptake ratios: Significant differences in tumor accumulation between the QFP-targeted and the control tracer were detected at 6 h p.i., 18 h p.i., and 28 h p.i. (*p* < 0.05); (**B**) Specific tumor accumulation is expressed as fold change of targeted-to-nontargeted tracer as a function of time p.i. Prolonged retention of the targeted tracer and tumor washout of the control lead to the upward trend.

Biodistribution of the QFP-phage-DOTA-^64^Cu and control-phage-DOTA-^64^Cu tracers was examined in immunocompetent C57Bl6 mice bearing subcutaneously implanted Lewis lung carcinoma (LLC) tumors and the results were compared. The tumor and normal organ distribution properties of the radiolabeled tracers at 2 h, 6 h, 18 h, and 28 h p.i. are summarized in Figure 2 and Table 1. *In vivo* kinetics revealed tumor-to-muscle (T/M) radioactive uptake ratio for the targeted tracer of 4.10 ± 1.56 at 2 h p.i., 7.33 ± 0.65 at 6 h p.i., 5.1 ± 0.57 at 18 h p.i., and 4.03 ± 1.47 at 28 h p.i. The tumor-to-muscle (T/M) ratios for the control nontargeted tracer were 4.65 ± 0.64, 4.00 ± 2.55, 2.57 ± 1.40, and 1.53 ± 1.37 at 2 h p.i., 6 h p.i., 18 h p.i., and 28 h p.i., respectively. Significant differences between the targeted and the control tracer were detected at 6 h p.i., 18 h p.i., and 28 h p.i. indicating the ability of QFP-peptide to specifically bind to tumor tissue and contribute to tumor retention of the radioactivity from the QFP-tracer. The tumor-to-muscle ratio for the QFP-tracer peaked at 6 h p.i. (7.33 ± 0.65). In contrast, the tumor-to-muscle ratio for the control continuously diminished with time post-injection, indicating a significant tumor washout rate. Furthermore, tumor accumulation of the targeted compared to nontargeted tracer increased with time p.i. (Figure 2B). Notably, the QFP-tracer yielded 83% more tumor accumulation than the control tracer at 6 h p.i., 99% more accumulation at 18 h p.i., and 163% more accumulation at 28 h p.i. These data provide evidence for the specific binding and retention of the QFP-tracer to s.c. LLC tumors in C57Bl6 mice. 

**Table 1:** Biodistribution of QFP-phage-DOTA-^64^Cu tracer and control-phage-DOTA-^64^Cu tracer in C57Bl6 mice bearing Lewis lung carcinoma tumors subcutaneously implanted in the inguinal region. Data are presented as the organ-to-muscle uptake ratios (mean ± SD, n = 3). * indicates statistical significance compared to the nontargeted control (*p* < 0.05).

Organ	2 h p.i.	6 h p.i.	18 h p.i.	28 h p.i.
QFP-tracer				
Tumor	4.10 ± 1.56	7.33 * ± 0.65	5.1 * ± 0.57	4.03 * ± 1.47
Liver	94.80 ± 23.19	58.77 ± 20.91	48.10 ± 15.70	38.43 ± 20.88
Lung	14.05 ± 3.61	10.40 ± 3.49	8.85 ± 0.21	6.20 ± 4.91
Kidney	19.95 ± 0.07	17.67 ± 8.14	11.95 ± 2.90	7.93 ± 2.22
Heart	4.55 ± 0.35	4.53 ± 0.87	4.53 ± 0.87	3.10 ± 0.95
Blood	6.02 ± 3.37	5.28 ± 2.15	3.97 ± 2.52	3.7 ± 0.28
Control-tracer				
Tumor	4.65 ± 0.64	4.00 ± 2.55	2.57 ± 1.40	1.53 ± 1.37
Liver	108.45 ± 16.90	82.37 ± 23.29	19.23 ± 8.77	24.43 ± 1.27
Lung	10.80 ± 0.14	10.20 ± 1.23	4.77 ± 2.91	6.6 ± 0.49
Kidney	16.95 ± 2.47	14.57 ± 1.46	10.73 ± 2.35	11.57 ± 0.29
Heart	3.65 ± 0.49	4.43 ± 0.78	3.70 ± 0.79	4.37 ± 1.20
Blood	4.83 ± 0.95	4.82 ± 1.36	3.72 ± 3.23	4.75 ± 0.35

As seen in Table 1, normal organ uptake of QFP- and control tracer was low with the exception of the liver. High uptake in the liver for both tracers can be explained by hepatobiliary excretion. This observation is consistent with findings from other groups who reported that phage and phage analogs are cleared by receptor mediated endocytosis in the liver [37]. There was very little radioactive retention in the remaining other vital organs including the heart. Based on this biodistribution profile, the QFP-tracer is not anticipated to cause any cardio-toxicity.

### 2.3. Small Animal PET/CT Imaging

**Figure 3:**
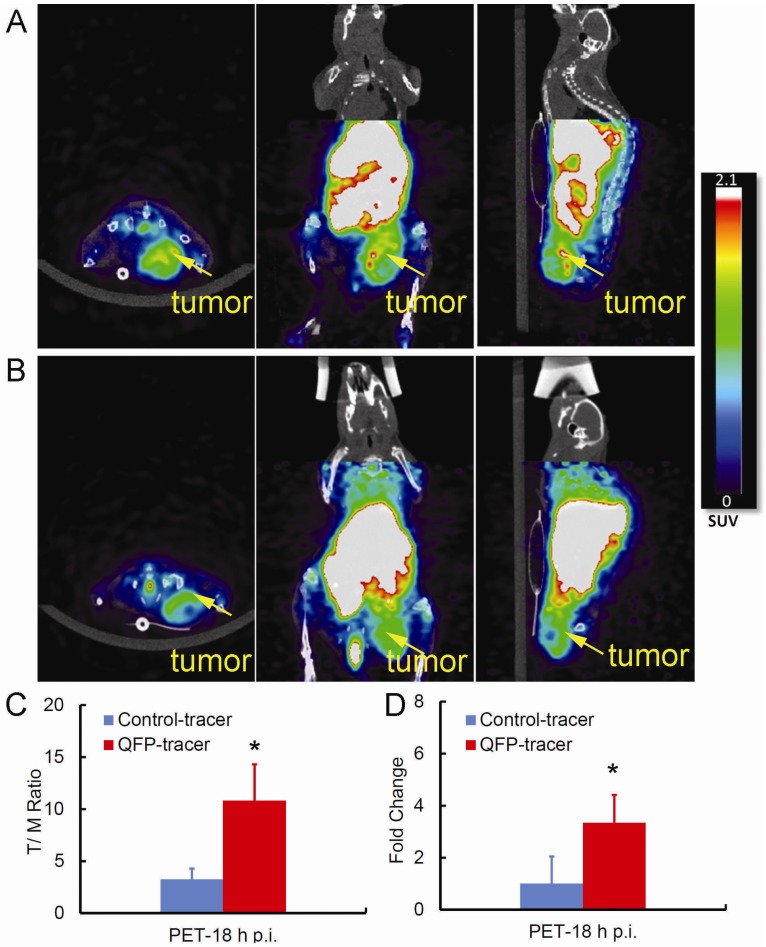
Ability of the QFP-tracer to specifically accumulate in tumor tissue based on PET/CT imaging. Twenty minute whole body static PET scans of C57Bl6 mice bearing s.c. LLC tumors were taken 18 h p.i. (**A**) Representative images using the QFP-tracer, and (**B**) the control tracer are displayed; (**C**) Quantification of the tumor-to muscle (T/M) uptake as determined by the ratio of tumor SUV-to-muscle SUV (*p* < 0.05); (**D**). Specific tumor accumulation expressed as fold change of tumor SUV for the QFP-targeted and tumor SUV for the control tracer (*p* < 0.05).

The tumor targeting properties of QFP-phage-DOTA-^64^Cu and the control-phage-DOTA-^64^Cu tracers were further evaluated in s.c. LLC tumors in C57Bl6 mice by noninvasive PET/CT imaging. A whole body PET/CT scan was conducted 18 h p.i. (Figure 3A,B). At 18 h after injection, radioactivity from blood had cleared, resulting in low levels of background activity and improved image contrast. Indeed the tumor was clearly visualized with the targeted tracer as was the liver which is consistent with the biodistribution studies (Figure 2 and Table 1). On the other hand, the control nontargeted radiotracer accumulated less in the tumor. As shown in Figure 3C differences in tumor accumulation of the QFP-targeted and the control radiotracer, quantified by T/M standardized uptake values (SUVs), were statistically significant which is in good agreement with the data obtained from direct tissue sampling. At 18 h p.i. the specificity of the QFP-tracer determined as the ratio of the tumor SUV for the QFP- and the control tracers was more than 3-fold greater (see Figure 3D). Together, these data confirm that binding of the QFP-peptide displayed on the surface of the phage scaffold has a direct influence on the tumor accumulation and retention of the QFP-tracer.

A common approach to establish specificity of a targeted vector is to conduct a PET blocking study via pre- or co-administration of nonradiolabeled peptide together with the radiotracer. Because the twelve amino acid QFP-sequence has circulation half-life of only several minutes and the large QFP-phage-DOTA-^64^Cu particle has orders of magnitude slower clearance kinetics the traditional approach for determining specificity of binding to a target on a molecular level is not applicable here. Instead we used a control with matched pharmacokinetic properties, *i.e.*, the wild type – phage – DOTA - ^64^Cu construct, and compared tumor-to-background ratios for the targeted and nontargeted vehicle (Figure 2 and Figure 3) to establish specificity on a tissue level.

In the present study PET imaging was performed 18 h after injection of QFP-phage-DOTA-^64^Cu tracer. However, favorable tumor-to-muscle ratios were already achieved at 6 h p.i. (Table 1 and Figure 2). Because visualization of angiogenic LLC tumors will depend both on tumor-to-background ratios and the ^64^Cu radioactive decay characteristics, the optimal time for imaging with this new tracer will be determined by multiple factors. We took advantage of the decay properties of the^64^Cu radionuclide to allow for delayed imaging with potentially improved image contrast and carried out the imaging study at 18 h p.i. Indeed the PET images of s.c. LLC tumor taken at 18 h p.i. reveal focal sites of radioactivity uptake and retention with relatively high resolution and background contrast. Also the micro PET images were able to discriminate necrotic regions of the tumor demonstrating the utility of this tracer to image very fine structures *in vivo*.

Noninvasive PET/CT imaging and direct tumor sampling independently provided evidence of ability of the QFP-tracer to bind tumor tissue specifically. In addition, PET/CT images showed that uptake of the QFP-phage-DOTA-^64^Cu in the tumor was heterogeneous and nonuniform. Heterogeneous tracer distribution within the tumor explains the observation that the magnitude for the T/M ratio for the targeted tracer calculated by the standardized uptake values is higher than the corresponding ratio determined from the tissue distribution study. Molecular PET provides information on a sub-tissue level, preferably details on cellular and molecular events, while the radioactivity values measured from the explanted tumor are averaged across the tumor mass. Thus in addition to being noninvasive in nature compared to *ex vivo* biopsy sampling, PET provides more accurate sub-tissue detail characterization of large heterogeneous tumors. 

From the results of the PET studies performed here, it follows that QFP-tracer would be appropriate for imaging lower abdomen. If major metastatic sites such as the liver and the GI tract are of interest, the entire QFP-conjugate should be re-designed for fast renal clearance with minimized liver accumulation.

### 2.4. *Ex-Vivo* Analysis of Intra-Tumoral Tracer Distribution

**Figure 4:**
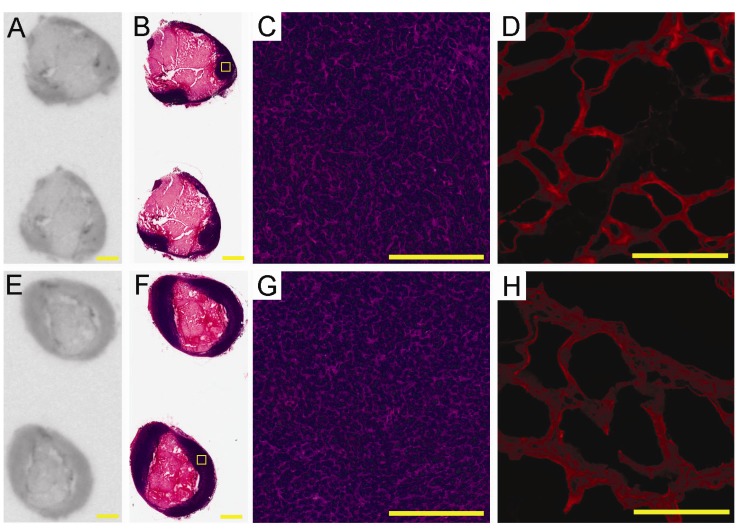
Representative images from *ex vivo* electronic autoradiography of regional tracer distribution (**A** and **E**), H & E histology (**B**, **C**, **F**, and **G**), and tumor blood vessels identified by lectin staining (**D** and **H**). Top row: distribution of the QFP-tracer; Bottom row: distribution of the control tracer. Scale bar on panels **A**, **B**, **E**, and **F** is 2 mm; Scale bar on panels **C**, **D**, **G**, and **H** is 200 µm. The yellow squares on panels **B** and **F** indicate areas which are magnified and presented on panels **C** and **G**, respectively.

To explore on a microscopic level the biodistribution patterns of the QFP-tracer within the tumor mass, we performed *ex vivo* autoradiography followed by histology and blood vessel visualization. After completion of the PET scan the tumor was retrieved, sectioned, and prepared for autoradiography, H & E histology, and lectin staining. Shown in Figure 4A,E are representative tumor sections from the autoradioagraphic analysis revealing the distribution patterns of the QFP- and the control tracer at 18 h p.i. Histology of the corresponding tumor sections is presented in Figure 4B,F. The tracers primarily localize at the edge of the tumors with no accumulation in the center of the tumor mass. H & E staining confirmed that the centers are necrotic. The morphological components of the LLC tumors in C57Bl6 mice observed here are in agreement with findings from Tanaka *et al.* [38], who reported a large necrotic core surrounded by viable LLC cells. Histologic sections at higher magnifications are presented in Figure 4C,G. Blood vessel networks identified by lectin staining are displayed in Figure 4D,H. Visual analysis indicates that the highly vascularized regions on the tumor edge seem to contain the highest tracer uptake. Detailed studies are underway to determine localization of the QFP-tracer to specific cellular population(s) within the tumor microenvironment. Additionally, the molecular target to which the QFP-peptide binds remains to be determined. Future studies are therefore warranted to identify and characterize biological events underlying molecular imaging of tumor neovasculature with the QFP-tracer.

### 2.5. Dosimetry Calculations

Human-absorbed dose estimates extrapolated from the rodent data were based on the biodistribution of the QFP-tracer in C57Bl6 mice obtained by direct tissue sampling at different time points. The absorbed activity for a given organ per hour and the MIRD S-value for ^64^Cu [39] were used to estimate human absorbed doses. Because most of the radiation dose from ^64^Cu is due to emission of positrons, the S-value assumption that positrons are locally absorbed within each organ (*i.e.*, with no contribution from cross-organs) was made. Results from the dosimetry analysis for liver, kidney and lung are summarized in Table 2. The absorbed radiation doses represent the normal dose per unit injected activity to the normal organs of an adult human male. Based on these data, the liver appears to be the dose-limiting organ with an average radiation dose of 0.641 Rad/mCi (0.173 mGy/MBq). This relatively high dose absorbed by the liver poses a potential concern. High liver accumulation of radioactivity reflects clearance patterns associated with the large phage particle [37] but also may be a consequence of limited *in vivo* stability of the ^64^Cu-DOTA complex. Under physiologic conditions, ^64^Cu may transchelate from the DOTA ligand and may become associated with copper binding proteins such as superoxide dismutase which can explain the relatively high liver uptake. Alternative chelator entities that form stable *in vivo* complexes with ^64^Cu represent a viable option to design QFP-based tracer with improved biodistribution and dosimetry profile.

**Table 2:** Human-absorbed radiation dose estimates resulting from administration of QFP-phage-DOTA-^64^Cu extrapolated from the mouse biodistribution data.

Organ	Organ uptake µCi.h/organ.mCi	S-factor Rad/µCi.h	Absorbed dose Rad/mCi (mGy/MBq)
Liver	3375	1.90E-04	0.641 (0.173)
Kidney	140	1.00E-03	0.140 (0.038)
Lung	222	3.00E-04	0.067 (0.018)

## 3. Experimental

### 3.1. Cell Culture, Animals and Tumors

Murine Lewis lung carcinoma (LLC) cell line was obtained from the American Type Culture Collection (Manassas, VA, USA). The LLC cells were grown in Dulbecco’s modified Eagles medium (DMEM) supplemented with 10% fetal bovine serum. Cells were maintained at 37 °C in a 5% CO_2_ humidified incubator. Sub-culturing was performed using standard trypsinization procedures.

Five to 6-week-old, immunocompetent C57BL/6 female mice were obtained from Jackson Laboratories, Inc. (Bar Harbor, ME, USA). The mice were supplied food and water *ad libitum* and were used within one month following initial acclimation. To establish tumors, mice were anesthetized by intraperitoneal administration of 0.2 M Avertin (approx. 0.2 mL per mouse). LLC cells were trypsinized, washed three times in DMEM, counted and re-suspended in 500 µL growth factor reduced Matrigel basement membrane matrix (BD Biosciences, Bedford, MA, USA). Approximately 1 × 10^6^ LLC cells were implanted subcutaneously via a 23-gauge needle in the right inguinal region of each mouse. Solid tumors were established over a period of seven days, resulting in mice with approximately 0.6 cm^3^-sized tumors for all experiments. 

Animal studies were conducted according to protocols approved by the Institutional Animal Care and Use Committee at the University of North Carolina at Chapel Hill and conform to the Guide for Care and Use of Laboratory Animals published by the National Institutes of Health (NIH Publication No. 85-23, revised 1996).

### 3.2. Radiotracer Synthesis

#### 3.2.1. Phage Propagation and Functionalization with DOTA

To obtain high titer stocks, QFP-phage or control wild type M13 phage were amplified in *E. coli* and purified by double precipitation with polyethylene glycol (PEG-8000) as described previously [36,40]. The phage titer was determined by a plaque assay. The isothiocyanate derivative of the macrocyclic bifunctional chelator 2-(4-isothiocyanato benzyl)-1,4,7,10 tetraazacyclododecane-1,4,7,10-tetraacetic acid (*p*-SCN-Bn-DOTA) (Macrocyclics, Dallas, TX, USA) was used for modifying M13 phage coat protein pVIII. To carry out this functionalization procedure, PEG-precipitated phage (1 × 10^12^ pfu) were re-suspended in 400 µL conjugation buffer (carbonate-bicarbonate buffer, pH adjusted to 9.0 with NaOH) and 4 µL p-SCN-Bn-DOTA (100 mM stock) added. The reaction mixture was incubated in a heat block at 35 °C overnight. Unbound DOTA was separated using 50 kDa Centiprep (YM-50) centrifugal filters (Millipore, Billerica, MA, USA) by centrifugation at 14,000 *g* for 10 min. For radiolabeling with ^64^Cu, QFP-phage-DOTA or control-phage-DOTA conjugates were re-suspended in 0.1 M sodium acetate buffer (pH 5.5) the titer was determined by a plaque assay, and solution concentration was adjusted to 2.5 × 10^11^ pfu/mL.

#### 3.2.2. Radiolabeling with ^64^Cu

High specific activity ^64^Cu (half-life t_1/2_ of ^64^Cu = 12.7 h, specific activity 14000 ± 7600 Ci/mmol or 518 ± 28 TBq/mmol) was obtained from the Washington University School of Medicine (St. Louis, MO, USA). ^64^Cu was produced on a CS-15 biomedical cyclotron by the ^64^Ni(p,n)^64^Cu nuclear reaction using previously published methods [41]. ^64^Cu in chloride solution was delivered overnight on the production date. Radiolabeling was performed at the UNC Small Animal Imaging Facility as follows: QFP-phage-DOTA (10^11^ pfu) or control phage-DOTA (10^11^ pfu) were radiolabeled with 2 mCi (74 MBq) ^64^Cu in sodium acetate buffer (0.1 M, pH 5.5) in a final volume of 400 µL at 50 °C for 50 min. Unreacted ^64^Cu was complexed by adding an aliquot of 2 mM EDTA (10 µL). The resulting radiolabeled conjugates were purified and concentrated by ultracentrifugation as described above. Both radioconjugates were obtained with radiolabeling efficiency of 98% or greater. QFP-phage-DOTA-^64^Cu or control-phage-DOTA-^64^Cu was reconstituted in sterile PBS (1 mCi/mL or 37 MBq/mL) and passed through a 0.22 µm filter immediately before injecting into the mice. 

### 3.3. Biodistribution Studies of ^64^Cu-Labeled Tracers in the s.c. LLC Tumor Model

Eight groups of mice (n = 3) bearing s.c. LLC tumors were injected intravenously via the tail vein with approximately 0.150 mCi (5.5 MBq) of the radiolabeled targeted or control tracer in a total volume of 150 µL in PBS. The tracer was allowed to circulate in the mice for 2 h, 6 h, 18 h, and 28 h. The mice were then sacrificed by cervical dislocation under anesthesia. The following tissues were collected, weighted, snap-frozen in liquid nitrogen, and counted in a WIZARD2 automated gamma counter (Perkin Elmer Life Sciences, Gaithesburg, MD): liver, kidney, lung, heart, skeletal muscle (quadriceps), and tumor. Uptake of radioactivity in the tumor and normal tissues per gram was normalized by the uptake of radioactivity of a gram of skeletal muscle and reported as the tumor-to-muscle ratio. 

### 3.4. Small Animal PET/CT Studies

#### 3.4.1. Experimental Protocol

MicroPET scans were performed on a small animal PET/CT scanner (eXplore Vista, GE Healthcare, Inc., Waukesha, WI, USA) with center resolution of 1.2 mm and a 46 mm axial field of view. At seven days post-implantation, mice bearing s.c. LLC tumors were injected with 0.5 mCi (18.5 MBq) QFP-phage-DOTA-^64^Cu or control-phage-DOTA-^64^Cu into the tail vein at a volume 150 µL (n = 3 per condition). Eighteen hours post-tracer injection, the animal was anesthetized using an isoflurane/air mixture (3% isoflurane for induction and 1.5% for maintenance), placed in a prone position on a supporting cradle, and advanced into the scanner. The animal’s respiratory rate was monitored by a respiratory probe placed underneath the belly, and body temperature was monitored through a rectal temperature probe during PET. A CT scan was first acquired for 7 min for subsequent attenuation correction and anatomical registration. All PET scans consisted of twenty minute static collection. After the scan was complete, the tumor was removed and snap-frozen in liquid nitrogen for autoradiography, histology, and fluorescence studies. 

#### 3.4.2. PET Image Analysis

Raw PET images were reconstructed using 2D ordered subset expectation maximization (OSEM) algorithms with scatter correction, random correction, and attenuation correction using the manufacturer proprietary software on the scanner console computer. A standardized uptake value (SUV) was calculated based on the calibrated counts, the injection dose, and animal body weight. Regions of interest (ROI) were drawn around the viable tumor area, *i.e.*, from the right lateral, actively perfused shell of the tumor, avoiding the very low or absent intensity regions which were presumed to be necrotic. The muscle ROI was selected in the uniform central region of the femur, placed away from the edges to avoid partial volume effect due to limited resolution in the PET images. 

### 3.5. Autoradiography, Histology and Fluorescence Imaging

The frozen tumor samples were cryo-sectioned at 14 µm thickness. For each tumor at least 4 consecutive slides were cut (2 for autoradiography and 2 for lectin staining) and processed using standard methods.

Electronic autoradiography was performed on a digital phosphor imager (Cyclone Plus, Perkin Elmer, Inc., Shelton, CT, USA). After cryo-sectioning the tissue, slides were exposed to a storage phosphor screen for one week at room temperature. The phosphor screen was then read in the phosphor imager system to visualize the intra-tumoral distribution of the targeted and the control tracer. After autoradiography, all slides were stained with haematoxylin and eosin as described previously [42]. To view the corresponding histology whole H & E sections were scanned on an Aperio Scanscope FL digital platform.

Blood vessels were visualized with TRITC-conjugated *Triticum vulgaris* lectin following protocols described previously [43]. Briefly, tumor sections were incubated in the dark with TRITC-lectin in PBS (50 mg/mL) for 1 h at room temperature. The samples were rinsed four times for 5 min in PBS and then one time for 5 min in distilled water. Sections were mounted with Prolong Gold reagent, and examined under an Olympus BX61 upright fluorescence microscope. Digital images were captured with a CCD camera. 

### 3.6. Dosimetry

Biodistribution data for the QFP-phage-DOTA-^64^Cu tracer in C57Bl6 mice obtained from direct tissue sampling were used for the dosimetry calculations. The assumption was made that the mouse biodistribution, determined as %ID/organ at various time points post-injection, is the same as the human distribution. Physical decay was assumed for activity remaining in organs beyond 28 h p.i. The organ values were decay-corrected using the appropriate decay constant, converted into µCi/organ/mCi, and plotted *versus* time. For each organ, the uptake in units µCi.h/organ/mCi was determined by measuring the area under the curve by the trapezoidal method. Human-absorbed dose estimates were calculated according to the Medical Internal Radionuclide Dose (MIRD) methodology by multiplying the organ uptake in µCi.h/mCi by the appropriate MIRD-provided S-values (Rad/µCi.h) for ^64^Cu distribution within each organ [39]. Absorbed radiation dose to the organs was calculated assuming homogeneous activity distribution throughout the organ. 

### 3.7. Statistical Analysis

All quantitative data are presented as mean ± standard deviation. For statistical analysis of the differences between the targeted and the control tracer, two-tailed Student’s unpaired t-test was performed. Differences were considered statistically significant at a value of *p* < 0.05. 

## 4. Conclusions

The results from this study indicate that in the context of the s.c. LLC mouse model, the QFP-peptide can target tumor blood vessels selectively. However, further optimization of the pharmacokinetic and dosimetry profile of the tracer is necessary to ensure efficient radiopharmaceutical applications enabled by the biological specificity of the QFP-peptide.

## References

[1] Rafii S., Lyden D., Benezra R., Hattori K., Heissig B. (2002). Vascular and haematopoietic stem cells: Novel targets for anti-angiogenesis therapy? Nat. Rev. Cancer.

[2] Lyden D., Hattori K., Dias S., Costa C., Blaikie P., Butros L., Chadburn A., Heissig B., Marks W., Witte L. (2001). Impaired recruitment of bone-marrow-derived endothelial and hematopoietic precursor cells blocks tumor angiogenesis and growth. Nat. Med..

[3] Duda D.G., Cohen K.S., Kozin S.V., Perentes J.Y., Fukumura D., Scadden D.T., Jain R.K. (2006). Evidence for incorporation of bone marrow-derived endothelial cells into perfused blood vessels in tumors. Blood.

[4] Aitsebaomo J., Srivastava S., Zhang H., Jha S., Wang Z.J., Winnik S., Veleva A.N., Pi X.C., Lockyer P., Faber J.E. (2011). Recombinant human interleukin-11 treatment enhances collateral vessel growth after femoral artery ligation. Arterioscler. Thromb. Vasc. Biol..

[5] Lyden D., Hattori K., Dias S., Hajjar K., Manova K., Moore M.A.S., Benezra R., Rafii S. (2000). Transplantation of bone marrow derived VEGF-responsive hematopoietic and vasculogenic precursor cells are essential to restore the angiogenic defect in Id1+/-Id3-/- knock out mice. Blood.

[6] Zhang H., Vakil V., Braunstein M., Smith E.L.P., Maroney J., Chen L., Dai K.Z., Berenson J.R., Hussain M.M., Klueppelberg U. (2005). Circulating endothelial progenitor cells in multiple myeloma: implications and significance. Blood.

[7] Furstenberger G., von Moos R., Lucas R., Thurlimann B., Senn H.J., Hamacher J., Boneberg E.M. (2006). Circulating endothelial cells and angiogenic serum factors during neoadjuvant chemotherapy of primary breast cancer. Br. J. Cancer.

[8] Dome B., Timar J., Dobos J., Meszaros L., Raso E., Paku S., Kenessey I., Ostoros G., Magyar M., Ladanyi A. (2006). Identification and clinical significance of circulating endothelial progenitor cells in human non-small cell lung cancer. Cancer Res..

[9] Rafat N., Beck G.C., Schulte J., Tuettenberg J., Vajkoczy P. (2010). Circulating endothelial progenitor cells in malignant gliomas Clinical article. J. Neurosurg..

[10] Roodhart J.M., Langenberg M.H., Vermaat J.S., Lolkema M.P., Baars A., Giles R.H., Witteveen E.O., Voest E.E. (2010). Late release of circulating endothelial cells and endothelial progenitor cells after chemotherapy predicts response and survival in cancer patients. Neoplasia.

[11] Duda D.G., Cohen K.S., di Tomaso E., Au A.P., Klein R.J., Scadden D.T., Willett C.G., Jain R.K. (2006). Differential CD 146 expression on circulating *versus* tissue endothelial cells in rectal cancer patients: Implications for circulating endothelial and progenitor cells as biomarkers for antiangiogenic therapy. J. Clin. Oncol..

[12] Pasqualini R., Ruoslahti E. (1996). Organ targeting *in vivo* using phage display peptide libraries. Nature.

[13] Hamzah J., Kotamraju V.R., Seo J.W., Agemy L., Fogal V., Mahakian L.M., Peters D., Roth L., Gagnon M.K.J., Ferrara K.W. (2011). Specific penetration and accumulation of a homing peptide within atherosclerotic plaques of apolipoprotein E-deficient mice. Proc. Natl. Acad. Sci. USA.

[14] Chen K., Ma W.H., Li G.Q., Wang J., Yang W.D., Yap L.P., Hughes L.D., Park R., Conti P.S. (2013). Synthesis and evaluation of Cu-64-labeled monomeric and dimeric NGR peptides for MicroPET imaging of CD13 receptor expression. Mol. Pharm..

[15] Chen K., Sun X.L., Niu G., Ma Y., Yap L.P., Hui X.L., Wu K.C., Fan D.M., Conti P.S., Chen X.Y. (2012). Evaluation of Cu-64 labeled GX1: A phage display peptide probe for PET imaging of tumor vasculature. Mol. Imaging Biol..

[16] Gronwall C., Stahl S. (2009). Engineered affinity proteins-Generation and applications. J. Biotechnol..

[17] Nilsson F.Y., Tolmachev V. (2007). Affibody (R) molecules: New protein domains for molecular imaging and targeted tumor therapy. Curr. Opin. Drug Discov. Dev..

[18] Tolcher A.W., Sweeney C.J., Papadopoulos K., Patnaik A., Chiorean E.G., Mita A.C., Sankhala K., Furfine E., Gokemeijer J., Iacono L. (2011). Phase I and pharmacokinetic study of CT-322 (BMS-844203), a targeted adnectin inhibitor of VEGFR-2 based on a domain of human fibronectin. Clin. Cancer Res..

[19] Skerra A. (2008). Alternative binding proteins: Anticalins-harnessing the structural plasticity of the lipocalin ligand pocket to engineer novel binding activities. FEBS J..

[20] Klevenz B., Butz K., Hoppe-Seyler F. (2002). Peptide aptamers: Exchange of the thioredoxin-A scaffold by alternative platform proteins and its influence on target protein binding. Cell. Mol. Life Sci..

[21] Silverman J., Lu Q., Bakker A., To W., Duguay A., Alba B.M., Smith R., Rivas A., Li P., Le H. (2005). Multivalent avimer proteins evolved by exon shuffling of a family of human receptor domains. Nat. Biotechnol..

[22] Steiner D., Forrer P., Pluckthun A. (2008). Efficient selection of DARPins with sub-nanomolar affinities using SRP phage display. J. Mol. Biol..

[23] Haubner R., Beer A.J., Wang H., Chen X.Y. (2010). Positron emission tomography tracers for imaging angiogenesis. Eur. J. Nucl. Med. Mol. Imaging.

[24] Nayak T.K., Brechbiel M.W. (2009). Radioimmunoimaging with longer-lived positron-emitting radionuclides: Potentials and challenges. Bioconjug. Chem..

[25] Anderson C.J., Jones L.A., Bass L.A., Sherman E.L.C., McCarthy D.W., Cutler P.D., Lanahan M.V., Cristel M.E., Lewis J.S., Schwarz S.W. (1998). Radiotherapy, toxicity and dosimetry of copper-64-TETA-octreotide in tumor-bearing rats. J. Nucl. Med..

[26] Lewis J.S., Lewis M.R., Cutler P.D., Srinivasan A., Schmidt M.A., Schwarz S.W., Morris M.M., Miller J.P., Anderson C.J. (1999). Radiotherapy and dosimetry of Cu-64-TETA-Tyr(3)-octreotate in a somatostatin receptor-positive, tumor-bearing rat model. Clin. Cancer Res..

[27] Rogers B.E., Bigott H.M., McCarthy D.W., Della Manna D., Kim J., Sharp T.L., Welch M.J. (2003). MicroPET imaging of a gastrin-releasing peptide receptor-positive tumor in a mouse model of human prostate cancer using a Cu-64-labeled bombesin analogue. Bioconjug. Chem..

[28] Prasanphanich A.F., Nanda P.K., Rold T.L., Ma L.X., Lewis M.R., Garrison J.C., Hoffman T.J., Sieckman G.L., Figueroa S.D., Smith C.J. (2007). Cu-64-NOTA-8-Aoc-BBN(7–14)NH2 targeting vector for positron-emission tomography imaging of gastrin-releasing peptide receptor-expressing tissues. Proc. Natl. Acad. Sci. USA.

[29] Gaertner F.C., Kessler H., Wester H.J., Schwaiger M., Beer A.J. (2012). Radiolabelled RGD peptides for imaging and therapy. Eur. J. Nucl. Med. Mol. Imaging.

[30] Dumont R.A., Deininger F., Haubner R., Maecke H.R., Weber W.A., Fani M. (2011). Novel Cu-64- and Ga-68-Labeled RGD conjugates show improved PET imaging of alpha(v)beta(3) integrin expression and facile radiosynthesis. J. Nucl. Med..

[31] Nielsen C.H., Kimura R.H., Withofs N., Tran P.T., Miao Z., Cochran J.R., Cheng Z., Felsher D., Kjaer A., Willmann J.K. (2010). PET Imaging of tumor neovascularization in a transgenic mouse model with a novel Cu-64-DOTA-knottin peptide. Cancer Res..

[32] Li W.P., Meyer L.A., Capretto D.A., Sherman C.D., Anderson C.J. (2008). Receptor-binding, biodistribution, and metabolism studies of (64)Cu-DOTA-cetuximab, a PET-imaging agent for epidermal growth-factor receptor-positive tumors. Cancer Biother. Radiopharm..

[33] Cai W.B., Wu Y., Chen K., Cao Q.Z., Tice D.A., Chen X.Y. (2006). *In vitro* and *in vivo* characterization of Cu-64-labeled Abegrin (TM) a humanized monoclonal antibody against integrin alpha(v)beta(3). Cancer Res..

[34] Cai W.B., Ebrahimnejad A., Chen K., Cao Q.Z., Li Z.B., Tice D.A., Chen X.Y. (2007). Quantitative radioimmunoPET imaging of EphA2 in tumor-bearing mice. Eur. J. Nucl. Med. Mol. Imaging.

[35] Philpott G.W., Schwarz S.W., Anderson C.J., Dehdashti F., Connett J.M., Zinn K.R., Meares C.F., Cutler P.D., Welch M.J., Siegel B.A. (1995). RadioimmunoPET: Detection of colorectal carcinoma with positron-emitting copper-64-labeled monoclonal antibody. J. Nucl. Med..

[36] Veleva A.N., Nepal D.B., Frederick C.B., Schwab J., Lockyer P., Yuan H., Lalush D.S., Patterson C. (2011). Efficient *in vivo* selection of a novel tumor-associated peptide from a phage display library. Molecules.

[37] Deutscher S.L. (2010). Phage display in molecular imaging and diagnosis of cancer. Chem. Rev..

[38] Tanaka T., Furukawa T., Fujieda S., Kasamatsu S., Yonekura Y., Fujibayashi Y. (2006). Double-tracer autoradiography with Cu-ATSM/FDG and immunohistochemical interpretation in four different mouse implanted tumor models. Nucl. Med. Biol..

[39] (1975). MIRD Pamphlet No. 11..

[40] Veleva A.N., Cooper S.L., Patterson C. (2007). Selection and initial characterization of novel peptide ligands that bind specifically to human blood outgrowth endothelial cells. Biotechnol. Bioeng..

[41] Kume M., Carey P.C., Gaehle G., Madrid E., Voller T., Margenau W., Welch M.J., Lapi S.E. (2012). A semi-automated system for the routine production of copper-64. Appl. Radiat. Isot..

[42] Yuan H., Schroeder T., Bowsher J.E., Hedlund L.W., Wong T., Dewhirst M.W. (2006). Intertumoral differences in hypoxia selectivity of the PET imaging agent Cu-64(II)-diacetyl-bis(N-4-methylthiosemicarbazone). J. Nucl. Med..

[43] Willis M.S., Dyer L.A., Ren R., Lockyer P., Moreno-Miralles I., Schisler J.C., Patterson C. (2012). BMPER regulates cardiomyocyte size and vessel density *in vivo*. Cardiovasc. Pathol..

